# Height and Risk of Hip Fracture: A Meta-Analysis of Prospective Cohort Studies

**DOI:** 10.1155/2016/2480693

**Published:** 2016-10-12

**Authors:** Zhihong Xiao, Dong Ren, Wei Feng, Yan Chen, Wusheng Kan, Danmou Xing

**Affiliations:** Department of Orthopaedics, Pu Ai Hospital of Tongji Medical College, Huazhong University of Science and Technology, 473 Hanzheng Street, Qiaokou District, Wuhan 430030, China

## Abstract

The association between height and risk of hip fracture has been investigated in several studies, but the evidence is inconclusive. We therefore conducted this meta-analysis of prospective cohort studies to explore whether an association exists between height and risk of hip fracture. We searched PubMed and EMBASE, Web of Science, and the Cochrane Library for studies of height and risk of hip fracture up to February 16, 2016. The random-effects model was used to combine results from individual studies. Seven prospective cohort studies, with 7,478 incident hip fracture cases and 907,913 participants, were included for analysis. The pooled relative risk (RR) was 1.65 (95% confidence interval (CI): 1.26–2.16) comparing the highest with the lowest category of height. Result from dose-response analysis suggested a linear association between height and hip fracture risk (*P*-nonlinearity = 0.0378). The present evidence suggests that height is positively associated with increased risk of hip fracture. Further well-designed cohort studies are needed to confirm the present findings in other ethnicities.

## 1. Introduction

Hip fracture is the femoral fracture that occurs between the articular of hip joint and 5 cm below the distal point of the lesser trochanter [1]. As a major part of osteoporotic fractures, hip fracture is a major cause of disability and functional impairment [2] and contributes to both morbidity and mortality in the elderly [3]. Approximately 1.66 million hip fractures occurred worldwide in 1990 and estimates suggest that this figure will rise to 4.5 million by the year 2050 [4, 5]. As the magnitude of this public health challenge is increasing, identification of risk factors for hip fracture becomes a salient public health priority and can help to better understand the pathogenesis of hip fractures.

Previous studies have shown that smoking, physical inactivity, and low body mass index were associated with increased risk of hip fracture [6–8]. Other potential risk factors like height has also been reported by several epidemiological studies. However, the relationship between height and risk of hip fracture is inconsistent. Some studies [9–15] have reported significantly increased risk among taller participants, while others [16, 17] have failed to confirm this finding. We therefore conducted a meta-analysis to evaluate the evidence from prospective cohort studies to explore whether an association exists between height and risk of hip fracture.

## 2. Materials and Methods 

### 2.1. Search Strategy

We conducted this meta-analysis according to the checklist of the Meta-Analysis of Observational Studies in Epidemiology [18]. A literature search (up to February 16, 2016) of PubMed and EMBASE, Web of Science, and the Cochrane Library for prospective cohort studies examining the association between height and risk of hip fracture was performed without language restriction. The search terms used were hip fracture and height. Pertinent studies were retrieved by further screening of the reference lists.

### 2.2. Study Selection

Studies were included for analysis if they met the following criteria: (1) having a prospective cohort design, (2) considering the association between height and risk of hip fracture, (3) reporting risk estimates with 95% confidence intervals (CIs) for at least three quantitative height categories (usually divided into tertile or quartile). Animal studies, reviews, conference abstracts, editorials, and letters were excluded. If a study was reported in more than one article, only the result with the longest follow-up years was used.

### 2.3. Data Extraction and Quality Assessment

Two reviewers (W. F. and Y. C.) independently carried out the data extraction. The following data were extracted from each included study: name of the first author, publication year, study name, study location, follow-up years, characteristics of participants (sex and age), number of cases and participants, fracture ascertainment, variables adjusted for in the analysis, and the relative risks (RRs) or hazard ratios (HRs) or odds ratios (ORs) of hip fracture and corresponding 95% CIs for all categories of height.

The Newcastle-Ottawa Scale [19] was used to evaluate study quality. This scale awards a maximum of nine points to each study: selection of the study groups (maximum 4 points), comparability of the study populations (maximum 2 points), and ascertainment of the outcome of interest (maximum 3 points). Studies that scored 0–3, 4–6, and 7–9 were considered as low, moderate, and high quality, respectively.

### 2.4. Statistical Analysis

To take into account heterogeneity between studies, a random-effects model [20] was used to calculate summary RRs and 95% CI for the highest versus the lowest categories of height and for the dose-response analysis. The hazard ratios (HRs) were considered equivalent to RRs. For study that reported RRs based on sex or different age groups, we pooled these RRs with inverse variance weight and used combined estimates for that study. The method proposed by Greenland and Longnecker [21] was used to compute study-specific slopes (linear trends) and 95% CIs from the natural logs of the RRs and CIs across categories of height. As this method requires that the distribution of cases and person-years or noncases and the RRs with the variance estimates for at least 3 quantitative exposure categories are known, we estimated the distribution of cases or person-years in studies that did not report these but reported the total number of cases/person-years [22].

We assigned the median or mean height in each category to the corresponding RR for each study. When the median or mean height per category was not reported, the midpoint of the upper and lower boundaries was considered the height of each category. If the lower or upper boundaries for the lowest and highest category were not available, we assumed the length of these categories to be the same as the closest category. We examined a potential nonlinear relationship between height and hip fracture risk by using restricted cubic splines with 3 knots at percentiles (10%, 50%, and 90%) of the distribution [23]. A *P* value for nonlinearity was calculated by testing the null hypothesis that the coefficient of the second spline was equal to zero [24].

Heterogeneity between studies was assessed by using *Q* statistics (significance level of *P*≦0.10) and *I* ^2^ statistics [25]. *I* ^2^ values of 25%, 50%, and 75% were considered to be low, moderate, and high degrees of heterogeneity, respectively [26]. Subgroup analyses stratified by sex, study location, number of participants and cases, follow-up years, quality scores, and adjustment for confounders were conducted to investigate sources of heterogeneity. We also performed sensitivity analysis to explore the effect of each individual study on the pooled result. Potential publication bias was examined by Begg's funnel plots and Egger's test [27, 28]. The trim and fill method was used to further assess the possible effect of potential publication bias on the overall result [29]. This method considers the possibility of hypothetical missing studies that might exist, estimates their RRs, and recalculates a pooled RR that incorporates the estimated RRs of these hypothetical missing studies. All statistical analyses were done using Stata version 11.0. *P* values were two-sided and *P* < 0.05 was considered statistically significant unless where otherwise stated.

## 3. Results

### 3.1. Literature Search

**Figure 1:**
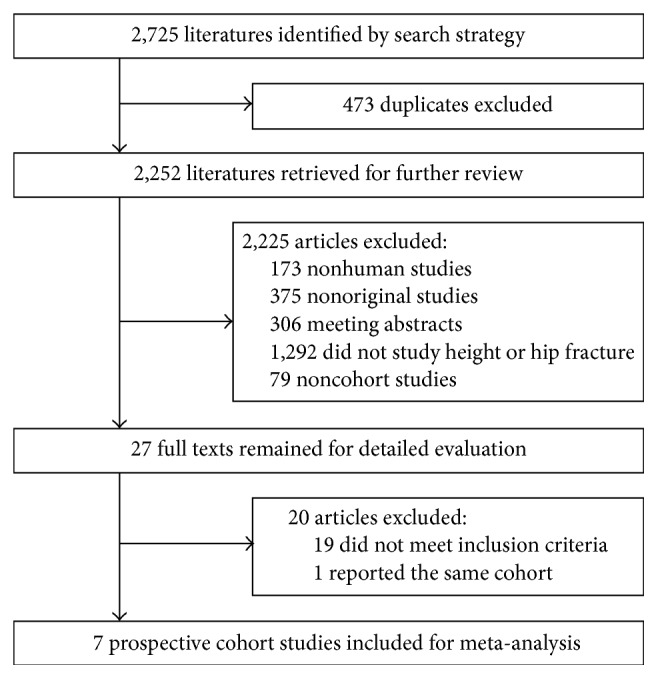
Flow diagram of the selection of prospective cohort studies.

Figure 1 presents the results of the literature search and study selection process. The initial search yielded 2,725 records using the search strategy: of these, 473 duplicate articles were removed resulting in retaining 2,252 abstracts for further review. Further, 2,225 articles were excluded during abstract screening (173 nonhuman studies, 375 nonoriginal studies, 306 meeting abstracts, 1,292 not studying height or hip fracture, and 79 not cohort studies). After evaluating the full text of the remained 27 articles, we excluded 19 articles that did not fulfill the inclusion criteria; we further excluded 1 article [30] that reported the same cohorts with short follow-up years with another article [10]. Finally, 7 prospective cohort studies [10–16] were included for analysis.

### 3.2. Study Characteristics

Table 1 shows the characteristics of the included studies. All the studies were prospective cohort studies published between 1991 and 2011, with a total of 907,913 participants with 7,478 incident hip fracture cases involved. Four studies were conducted in the US [10, 13–15]; the other 3 were conducted in Europe [11, 12, 16]. Four studies [11, 12, 15, 16] consisted of both women and men, 2 studies [10, 13] only involved women, and 1 study [14] only consisted of men. The age of participants at baseline varied from 35 to more than 85 years old. The follow-up years ranged from 7 years to 22 years. All included studies reported adjusted RR, and the potential confounding factors being adjusted for varied in different studies, including age, alcohol consumption, smoking, weight, and education. For the study quality assessment result, 2 studies [10, 15] were in moderate quality while other 5 studies [11–14, 16] were in high quality (see Table S1 in Supplementary Material available online at http://dx.doi.org/10.1155/2016/2480693).

### 3.3. Tall versus Short

**Figure 2:**
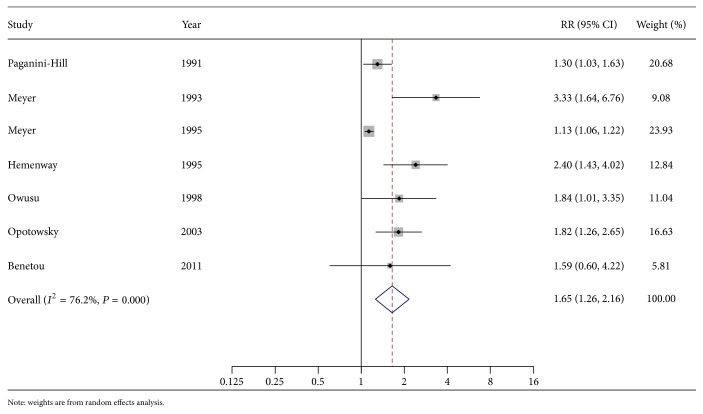
A forest plot of the association between height and risk of hip fracture.

Figure 2 shows a forest plot presenting the association between height and risk of hip fracture. The summary RR of hip fracture for the highest versus lowest category in height was 1.65 (95% CI: 1.26 to 2.16). Statistically significant heterogeneity was observed among the individual results (*I* ^2^ = 76.2%, *P*-heterogeneity < 0.05).

### 3.4. Dose-Response Meta-Analysis

**Figure 3:**
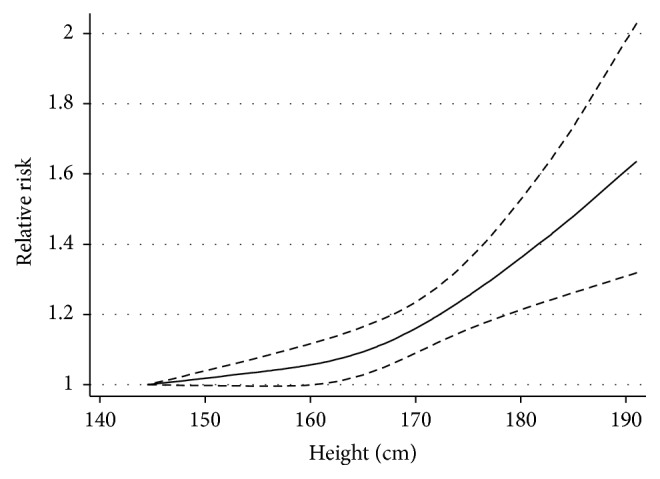
Dose-response relations between height and relative risk of hip fracture. The solid line and the long dashed lines represent the estimated relative risk and corresponding 95% CI, respectively. There was evidence of a nonlinear association between height and hip fracture risk (*P*-nonlinearity = 0.0378).

Figure 3 presents the dose-response relations between height and relative risks of hip fracture. There was evidence of a nonlinear association between height and hip fracture risk (*P*-nonlinearity = 0.0378). As compared with individuals who were in the lowest height, the RR of hip fracture for each 10 cm increment was 1.007 (95% CI: 1.002–1.012).

### 3.5. Publication Bias

Publication bias was found across the included studies indicated by Begg's and Egger's test (both *P* values < 0.05). However, the trim and fill method identified 3 hypothetical missing studies, and the repooled results incorporating the hypothetical studies continued to show a statistically significant association between height and risk of hip fracture (RR = 1.34, 95% CI: 1.06–1.71).

### 3.6. Subgroup Analysis and Sensitivity Analysis

Table 2 summaries the results of subgroup analysis according to study characteristics. The associations between height and hip fracture risk were similar in subgroup analyses stratified by sex, study location, number of participants or cases, follow-up years, quality scores, and adjustment for confounders. Sensitivity analysis showed that the summary RR was not substantially influenced by any of the individual study, with a range from 1.50 (95% CI: 1.18–1.92) when omitting Meyer et al.'s (1993) study [11] to 1.83 (95% CI: 1.38–2.44) when excluding Meyer et al.'s (1995) study [12].

## 4. Discussion

Based on the meta-analysis of 907,913 participants from prospective cohort studies, the present study found a positive association between height and increased risk of hip fracture. A nonlinear association between height and the risk of hip fracture was observed in the dose-response analysis.

Several plausible mechanisms exist for the association between height and risk of hip fracture. First, the center of gravity of taller people was higher than that of shorter people; thus taller people have further probability to fall and may hit the ground with more energy when falling [13]. Second, hip axis length, which refers to the distance from the lower base of the greater trochanter to inner pelvic brim [31], is suggested to be positively associated with increased risk of hip fracture, even after controlling for age, weight, and BMD [31, 32]. However, it has been shown that height and hip axis length are highly correlated, with an estimated correlation coefficient as high as more than 0.5 in different ethnic groups [33]. This implies that taller people might have higher risk of hip fracture due to the longer hip axis length. Third, growth is determined by both genetic and environmental factors, such as dietary intake, living conditions, and physical activities [34]. However, the environmental factors that related to height may act synchronously to the increased risk of hip fracture.

Hip fracture affects both men and women. In our study, a greater summary HR of hip fracture was observed in women (HR = 1.60, 95% CI: 1.18–2.16) than in men (HR = 1.42, 95% CI: 1.00–2.02). However, because the CIs for HR of hip fracture are overlapping for women and men, and meanwhile the lower limit of the CI for men includes 1, whether there is a gender difference of association between body height and risk of hip fracture should be further explored.

Due to the aging of the population, the number of hip fractures continues to increase [35, 36]. Although it is possible for most patients to return to their function level before the fracture with surgery followed by early mobilization, the treatment costs may result in increased risk of morbidity and mortality for some patients [37]. It is therefore imperative that we identify risk factors for hip fracture, with the goal of directing primary prevention. Unlike diet and behavior factors, height is a nonmodifiable characteristic. Nevertheless, the findings of our study have significance for the identification of high-risk population.

Our meta-analysis had several strengths. Because this meta-analysis only included prospective cohort studies, we minimized the potential effect of recall bias on our findings. In addition, each included study had a follow-up period long enough to observe potential association between height and risk of hip fracture. Another strength was that the studies involved a large number of participants; it was therefore possible to detect moderate reductions in risk.

Our study also had some limitations that deserve consideration. First, because our meta-analysis was based on observational studies, we could not rule out potential confounding from other fracture risk factors. Although the included studies adjusted for several potential confounders, for example, all studies adjusted for age, three studies adjusted for alcohol intake, and three studies adjusted for smoking, the association between height and risk of hip fracture could potentially be due to residual confounding from other factors related to tall people.

Second, although it has been shown that self-report was relatively accurate for hip fracture in adults [38, 39], however, there might be also misclassification of height and hip fracture due to less valid self-reported height in the elderly. Underestimation of height might be related to the height loss in old people who reported their height as measured in early adulthood without being aware of changes in their stature [40].

Third, the potential publication bias existed across the included studies indicated that studies reporting positive association between height and risk of hip fracture were more easily published, as indicated by Egger's test and Begg's test. However, when we performed the trim and fill method, the result continued to suggest statistically significant association between height and hip fracture, although the pooled RR was attenuated by the hypothesized missing studies.

Fourth, high statistical heterogeneity was found across the studies. One potential source of heterogeneity might be the difference of the number of hip fracture cases between studies, as suggested by the subgroup analysis. Although the heterogeneity may weaken the strength of our findings, the subgroup analysis and sensitivity analysis showed that results from our meta-analysis were robust.

Finally, the results of our meta-analysis were based on studies that only involved participants from Europe or the United States. However, the genetic factors and environmental factors with respect to height varied among different ethnicities, especially between east and west. Therefore, the findings of this study should be interpreted with caution among the population from the east.

## 5. Conclusions

In summary, the results from this meta-analysis of prospective studies indicate that height is positively associated with risk of hip fracture. However, due to residual confounding from other fracture risk factors related to height, the findings from this meta-analysis need to be confirmed by well-designed prospective studies, especially among other ethnicities.

## Supplementary Material

Table S1 presented the methodological quality score of included studies assessed by the Newcastle-Ottawa Scale (NOS). Studies that scored 0–3, 4–6, and 7–9 were considered as low, moderate, and high quality, respectively. 5 studies were in high quality while the other 2 studies were in moderate quality.

## Figures and Tables

**Table 1:** Characteristics of epidemiological studies of height and risk of hip fracture included in the meta-analysis.

Study	Cohort	Age (years)	Number of cases/number of participants	Follow-up years	Endpoint ascertainment	Sex	Exposure level (cm)^2^	RR (95% CI)	Adjustment for confounders
Paganini-Hill et al., 1991, US. [15]	The Leisure World Study	Median 73	418/13,649	7	Medical records	Female	≧165.1 versus ≦157.5	1.26 (0.98, 1.62)	Age
						Male	≧180.3 versus ≦170.2	1.48 (0.86, 2.55)	

Meyer et al., 1993, Norway [11]	National Health Screening Service (1974–1990)	35–49	210/52,313	10.9	Medical records	Female	≧170 versus <155	3.62 (1.46, 8.97)	Age
						Male	≧185 versus <170	2.92 (0.94, 9.05)	

Meyer et al., 1995, Norway [12]	National Health Screening Service (1963–1975)	50–89	6,087/673,848	16.4	Death certificates	Female	A1: >165 versus ≦157	1.26 (0.74, 1.68)	Age
							A2: >163 versus ≦156	1.10 (0.96, 1.26)	
							A3: >161 versus ≦154	1.19 (1.04, 1.35)	
							A4: >160 versus ≦153	1.07 (0.81, 1.41)	
						Male	A1: >178 versus ≦170	0.98 (0.68, 1.42)	
							A2: >176 versus ≦168	1.20 (0.98, 1.45)	
							A3: >175 versus ≦167	1.10 (0.90, 1.33)	
							A4: >174 versus ≦165	1.00 (0.67, 1.51)	

Hemenway et al., 1995, US. [10]	Nurses Health Study	35–59	243/92,804	10	Self-report, confirmed by medical records	Female	≧172.7 versus 157.5	2.40 (1.43, 4.02)	Age, body mass index, smoking status, alcohol consumption

Owusu et al., 1998, US. [14]	Health Professionals Follow-Up Study	40–75	56/43,053	8	Self-report	Male	≧188 versus ≦168	3.97 (1.28, 12.3)	Age, weight, calcium intake, smoking, alcohol consumption, waist-to-hip ratio

Opotowsky et al., 2003, US. [13]	NHANES I Epidemiologic Follow-Up Study	40–74	203/4,264	22	Medical records	Female	B1: ≧168.3 versus ≦155.2	3.91 (1.37, 11.13)	Age, weight, age at menopause, hormone use, recreational activity, nonrecreational activity, alcohol use, history of fracture, history of chronic diseases
							B2: ≧165.4 versus ≦152.0	2.01 (1.18, 3.44)	
							B3: ≧165.4 versus ≦152.1	1.27 (0.70, 2.29)	

Benetou et al., 2011, 5 European countries^1^ [16]	EPIC-Elderly-NAH Study	60–86	261/27,982	8	Self-report or medical records	Female/male	≧180 versus ≦149	1.59 (0.60, 4.22)	Age, educational level, smoking status, history of diabetes mellitus

^1^Including Germany, Greece, Italy, Netherlands, and Sweden.

^2^A1: 50–59 years; A2: 60–69 years; A3: 70–79 years; A4: 80–89 years; B1: 40–59 years; B2: 60–69 years; B3: 70–74 years.

**Table 2:** Summary of the results on association between height and risk of hip fracture.

Variables	Number of studies	RR (95% CI)	*I* ^2^%	*P*
Overall	7	1.65 (1.26, 2.16)	76.2	0.000
Location				
US	4	1.69 (1.27, 2.24)	50.3	0.110
Europe	3	1.72 (0.83, 3.58)	78.5	0.000
Follow-up years				
<10	3	1.37 (1.11, 1.69)	0.0	0.543
≧10	4	1.88 (1.15, 3.07)	86.5	0.000
Sex				
Female	5	1.60 (1.18, 2.16)	78.8	0.000
Male	4	1.42 (1.00, 2.02)	50.9	0.106
Both sex	1	1.59 (0.60, 4.22)	NA	NA
Number of participants				
<50000	4	1.47 (1.22, 1.76)	0.0	0.402
≧50000	3	1.96 (0.96, 4.01)	88.0	0.000
Number of cases				
<400	5	2.07 (1.62, 2.64)	0.0	0.569
≧400	2	1.16 (1.04, 1.29)	23.6	0.252
Study quality				
Moderate	2	1.69 (0.93, 3.06)	77.9	0.034
High	5	1.71 (1.14, 2.56)	76.9	0.000
Adjustment for confounders				
Number of confounders				
<4	3	1.39 (1.01, 1.91)	80.0	0.007
≧4	4	1.94 (1.49, 2.51)	0.0	0.812
Alcohol				
Yes	3	1.97 (1.50, 2.57)	0.0	0.675
No	4	1.39 (1.04, 1.86)	71.1	0.016
Smoking				
Yes	3	2.06 (1.43, 2.96)	0.0	0.690
No	4	1.49 (1.11, 2.01)	80.7	0.001
Weight				
Yes	2	1.83 (1.33, 2.50)	0.0	0.976
No	5	1.58 (1.15, 2.17)	77.7	0.001
