# Supplementary material for: Height and Risk of Hip Fracture: A Meta-Analysis of Prospective Cohort Studies
Source: Biomed Res Int. 2016 Oct 12;2016:2480693. doi: 10.1155/2016/2480693 (PMC5080474; doi:10.1155/2016/2480693)
Supplement: Supplementary file 1 — Table S1 presented the methodological quality score of included studies assessed by the Newcastle-Ottawa Scale (NOS). Studies that scored 0–3, 4–6, and 7–9 were considered as low, moderate, and high quality, respectively. 5 studies were in high quality while the other 2 studies were in moderate quality. [file 2480693.f1.doc]

**Table S1.** Methodological quality of included prospective cohort studies

| First author | Representativeness of the exposed cohort | Selection of the unexposed cohort | Ascertainment of exposure | Outcome of interest not present at start of study | Control for important factor or additional factor^1^ | Outcome assessment | Follow-up long enough for outcomes to occur | Adequacy of follow-up of cohorts | Total quality scores |
| --- | --- | --- | --- | --- | --- | --- | --- | --- | --- |
| Paganini-Hill [15] | ☆ | ☆ | — | — | ☆ | ☆ | ☆ | ☆ | 6 |
| Meyer (1993) [11] | ☆ | ☆ | ☆ | — | ☆ | ☆ | ☆ | ☆ | 7 |
| Meyer (1995) [12] | ☆ | ☆ | ☆ | — | ☆ | ☆ | ☆ | ☆ | 7 |
| Hemenway [10] | — | ☆ | — | — | ☆☆ | ☆ | ☆ | ☆ | 6 |
| Owusu [14] | ☆ | ☆ | ☆ | ☆ | ☆☆ | — | ☆ | ☆ | 8 |
| Opotowsky [13] | ☆ | ☆ | ☆ | ☆ | ☆☆ | ☆ | ☆ | ☆ | 9 |
| Benetou [16] | — | ☆ | ☆ | — | ☆☆ | ☆ | ☆ | ☆ | 7 |

^1^ A maximum of 2 stars were assigned to this item.
